# Treatment of artificial wastewater containing two azo textile dyes by vertical-flow constructed wetlands

**DOI:** 10.1007/s11356-017-0992-0

**Published:** 2017-12-21

**Authors:** Amjad Hussein, Miklas Scholz

**Affiliations:** 1 Civil Engineering Research Group, School of Computing, Science and Engineering, The University of Salford, Newton Building, Peel Park Campus, Salford, Greater Manchester M5 4WT UK; 2 Present Address: Civil Engineering Department, Engineering College, Al-Muthanna University, Al-Muthanna Samawah, Iraq; 3 Division of Water Resources Engineering, Department of Building and Environmental Technology, Faculty of Engineering, Lund University, P.O. Box 118, 221 00 Lund, Sweden; 4 Department of Civil Engineering Science, School of Civil Engineering and the Built Environment, University of Johannesburg, Kingsway Campus, Auckland Park, P.O. Box 524, Johannesburg, 2006 South Africa

**Keywords:** Acid Blue 113, Basic Red 46, Chemical oxygen demand, Common reed, Environmental pollution control, Textile wastewater

## Abstract

The release of untreated dye textile wastewater into receiving streams is unacceptable not only for aesthetic reasons and its negative impacts on aquatic life but also because numerous dyes are toxic and carcinogenic to humans. Strategies, as of now, used for treating textile wastewaters have technical and economical restrictions. The greater part of the physico-chemical methods, which are used to treat this kind of wastewater, are costly, produce large amounts of sludge and are wasteful concerning some soluble dyes. In contrast, biological treatments such as constructed wetlands are cheaper than the traditional methods, environmental friendly and do not produce large amounts of sludge. Synthetic wastewater containing Acid Blue 113 (AB113) and Basic Red 46 (BR46) has been added to laboratory-scale vertical-flow construction wetland systems, which have been planted with *Phragmites australis* (Cav.) Trin. ex Steud. (common reed). The concentrations 7 and 208 mg/l were applied for each dye at the hydraulic contact times of 48 and 96 h. Concerning the low concentrations of BR46 and AB113, the unplanted wetlands are associated with significant (*ρ* < 0.05) reduction performances, if compared with planted wetlands concerning the removal of dyes. For the high concentrations of AB113, BR46 and a mixture of both of them, wetlands with long contact times were significantly (*ρ* < 0.05) better than wetlands that had short contact times in terms of dye, colour and chemical oxygen demand reductions. Regarding nitrate nitrogen (NO_3_-N), the reduction percentage rates of AB113, BR46 and a mixture dye of both of them were between 85 and 100%. For low and high inflow dye concentrations, best removals were generally recorded for spring and summer, respectively.

**Electronic supplementary material:**

The online version of this article (10.1007/s11356-017-0992-0) contains supplementary material, which is available to authorized users.

## Introduction

Textile dyeing processes are one of the most environmental-unfriendly industrial processes, because the reagents used are very rich in chemical compounds comprising both inorganic and organic products (Juang et al. 1996; Robinson et al. 2001). Furthermore, the presence of colour in the effluent textile wastewater is one of the most important problems. Sultana (2014) stated that coloured wastewaters, produced from dyeing processes, are heavily polluted with chemicals, textile auxiliaries and dyes. The properties of textile wastewater depend on the production, technology and chemicals used (Wang et al. 2011).

Textile industries devour gigantic amounts of water and generate vast volumes of wastewater through different steps in the dyeing and finishing processes, and the discharged wastewater is an overwhelming blend of various polluting substances such as organic, inorganic, elemental and polymeric products (Babu et al. 2007; Kant 2012). Dye wastes are the most dominating materials in textile wastewater, and these materials are often toxic to the biological world as well as the dark colour of some of these materials blocking sunlight, which causes acute problems in biological communities (Ratna and Padhi 2012; Dey and Islam 2015).

The use of constructed wetlands in azo textile dye wastewater treatment is still at an experimental stage (Nawab et al. 2016). Although many researchers investigated the performance of constructed wetlands to treat textile wastewater in terms of dye, chemical oxygen demand (COD), phosphorus and nitrogen reductions, all corresponding results related to short-term operation and the data rarely covered all seasons (Table 1).

**Table 1:** Previous studies (listed in order of date) on textile wastewater treatment by constructed wetlands

Dye used	Type of wetland	Design characteristics	Plants used	Removal performance	Duration (days)	Country of operation	References
AB113, *RB171	VF	Gravel-sand	*P. australis*	98% colour	70	USA	Pervez et al. (1999)
AO7	VF	Gravel-sandy clay soil	*P. australis*	74% colour, 64% COD and 71% TOC	77	Portugal	Davies et al. (2005)
AO7	VF	Gravel-sandy clay soil	*P. australis*	99% colour, 93% COD and TOC	48	Portugal	Davies et al. (2006)
Various dyes in real wastewater	HF	Gravel-sand	*Typha* and *cocoyam*	77% colour, 72% COD and 59% sulfate	84	Tanzania	Mbuligwe (2005)
**RB5, DY211, VY46	VF-HF	Gravel-sand-tuff	*P. australis*	90% colour, 84% COD, 93% TSS, 52% TN, 87% N_organic_, − 331% NH_4_-N, 88% sulfate, 80% anion surfactant and 93% TSS	60	Slovenia	Bulc and Ojstršek (2008)
RR22, VR13, **RB5	VF	Gravel-sand-zeolite-peat	Without plant	70% dye, 60% EC, 88% COD and TOC	90	Slovenia	Ojstršek et al. (2007)
AO7	UF	Gravel-glass beads	*P. australis*	98% dye, 90% COD, 67% TN, 28% TP, 98% NH_4_-N, 100% NO_3_-N	365^a^	Japan	Ong et al. (2010)
RR141	VF	Gravel-sand	*Typha*	49% colour, 60% COD, 86% TDS		Thailand	Nilratnisakorn et al. (2009)
AO7	VF	Gravel-sludge	*P. australis*	94% colour, 95% COD and 86% NH_4_-N	27	N/A	Ong et al. (2011)
	FWS-SSF	Shale	*P. australis*	98% COD, 97%, colour		Thailand	Cumnan and Yimrattanabovorn (2012)
	SSF-FWS			91% COD, 99% colour			
Mixture dyes into different metabolites	VF	Coconut shavings-soil with bacteria	*Gaillardia pulchella*	70% COD, 74% TOC, 70 BOD	0.042	India	Kabra et al. (2013)
Mixture dyes into different metabolites	VF	Coconut shavings-sand-gravel-soil with bacteria	*Portulaca grandiflora*	59% COD, 38% BOD, 37% TOC, 41% turbidity, 71% TDS, 60% TSS	0.05	India	Khandare et al. (2013)
Various dyes in real wastewater	VF	Coconut shavings-gravel-sand-soil	*Typha*	79% COD, 77% BOD, 59% TDS, 27% TSS	3	Pakistan	Shehzadi et al. (2014)
AY 2G E107	VF	Gravel-sand-zeolite	*Canna* and *Typha*	95% colour, 64% COD, 94% PO_4_-P, 77% NH_4_-N	90	Turkey	Yalcuk and Dogdu (2014)

*AB* acid blue, *RB reactive blue, *VF* vertical-flow, *AO* acid orange, *COD* chemical oxygen demand, *TOC* total organic carbon, *HF* horizontal flow, **RB reactive black, *DY* disperse yellow, *VY* vat yellow, *TSS* total suspended solid, *TN* total nitrogen, *N* nitrogen, *NH*_*4*_ *-N* ammonium nitrogen, *RR* reactive red, *VR* vat red, *EC* electrical conductivity, *UF* upper flow, *NO*_*3*_ *-N* nitrate nitrogen, *TDS* total dissolved solids, *FWS* free water surface, *SSF* subsurface flow, *BOD* biochemical oxygen demand, *AY* acid yellow, *PO*_*4*_ *-P* ortho-phosphate-phosphorus, *N/A* not applicable

^a^The experimental work was under control condition (indoor)

Two azo textile dyes [Acid Blue 113 (AB113) and Basic Red 46 (BR46)] were selected in this research with two different concentrations: low with a target concentration of 5 mg/l and high with a target concentration of 200 mg/l. Typically, textile industry-processing effluents contain dyes in the range between 10 and 200 mg/l (Lavanya et al. 2014). Most textile dyes can be detected at a rather low concentration of even < 1 mg/l by the human eye (Chung 1983; Lavanya et al. 2014; Pandey et al. 2007). Furthermore, Van der Zee (2002) stated that algal growth was not inhibited at dye concentrations < 1 mg/l. Both of which are commercial dyes, which are extensively used in the textile industry (Chung et al. 1992; Riu et al. 1997; Pervez et al. 1999; Olgun and Atar 2009; Ong et al. 2010; Deniz and Karaman 2011; Deniz and Saygideger 2011). AB113 is an acid dye, and BR46 is a basic dye. An acid dye is defined as a negatively charged dye at a chemical level, which contains one or more acidic groups such as a sulfonic group (Akbari et al. 2002; Martínez-Huitle and Brillas 2009). A basic dye is defined as a positively charged stain at a chemical level (Martínez-Huitle and Brillas 2009; Brillas and Martínez-Huitle 2015), which means it reacts well with negatively charged materials (Sun and Yang 2003).

The aim of this project is to evaluate the effectiveness of vertical-flow constructed wetlands in reducing azo textile dyes contaminated with artificial wastewater, aromatic amines and other water quality variables including COD and ortho-phosphate-phosphorus (PO_4_-P). The corresponding objectives are to assess (a) the role of plants in reducing azo textiles within artificial wastewater, (b) the influence of the mixture of both of these two dyes on the performance of vertical-flow constructed wetlands, (c) the ability of this type of constructed wetland to reduce aromatic amines and (d) the influence of seasonal variation and operational parameters such as resting and contact times on dye reduction.

## Materials and methods

### Wetland set-up and operation

The research has been performed between 1 June 2016 and 31 May 2017. This system has been used for treating azo textile dye wastewater since 1 May 2015 (Hussein and Scholz 2017). The constructed wetlands have been located within a university greenhouse (Supplementary Material 1) and operated to treat artificial wastewater treating two azo textile dyes. The rig consisted of 18 vertical-flow constructed wetlands. Wastewater drained vertically to enhance aerobic biodegradation of nitrogen and organic matter (Fuchs 2009). The experiment evaluates the wetland performance by simulating processes occurring within large-scale reed beds. The filters were located at random within the system set-up. Resting and contact times as well as hydraulic loading rate impacts on dye removal were evaluated. The period of time when a wetland is empty (no liquid inside) is known as resting time, while contact time is known as the duration of the wastewater when it is in touch with the aggregates and/or plants in the system.

In this study, artificial wastewater containing two azo dyes (BR46 and AB113) was assessed at the concentrations of 7 and 208 mg/l for the contact times of 48 and 94 h with respect to their impact on the constructed wetland performance. All artificial wastewater chemicals (Wießner et al. 2005; Ong et al. 2009) were bought from the Scientific Laboratory Supplies (Wilford Industrial Estate, Wilford, Nottingham, UK). Details of each dye and the composition of artificial wastewater including its chemical concentrations used in the experimental work are shown in Supplementary Material S1 and Table 2, respectively. BR46 has a maximum absorbance (*λ*_max_) of 530 nm (Khataee 2009) and was sourced from DyStar (Am Prime Park, Raunheim, Germany). AB113 had a *λ*_max_ of 566 nm (Shirzad-Siboni et al. 2014) and was purchased from Sigma-Aldrich (The Old Brickyard, New Road Gillingham, Dorset, UK). Both dyes were used without further purification. The wavelength for the maximum absorbance of the dye mixture had been determined experimentally by using a WPA Biowave II Spectrophotometer (Biochrom, Cambourne Business Park, Cambourne, Cambridge, UK). At first, *λ*_max_ of the mixed dye was determined by scanning the absorption of different dye mixture concentrations for wavelengths between 300 and 800 nm. The *λ*_max_ for the mixed dye was found to be 511 nm.

**Table 2:** Details of artificial wastewater compositions use in the experimental work

Material	Chemical structure	Molecular weight (g/mol)	CAS number	Purity of dye (%)	Concentration (mg/l)
Sodium acetate anhydrous pure	CH_3_COONa	82.03	127-09-3	≥ 99	107.1
Sodium benzoate	C_6_H_5_COONa	144.11	532-32-1	≥ 99	204.9
Ammonium nitrate pure	NH_4_NO_3_	80.04	6484-52-2	≥ 99	76.1
Sodium chloride pure	NaCl	58.44	7647-14-5	≥ 99	7.0
Magnesium chloride hexahydrate	MgCl_2_·6H_2_O	203.30	7791-18-6	≥ 99	3.4
Calcium chloride dehydrate	CaCl_2_·2H_2_O	147.01	10035-04-8	≥ 99	4.0
Potassium phosphate dibasic trihydrate	K_2_HPO_4_·3H_2_O	228.22	16788-57-1	≥ 99	36.7

*CAS* Chemical Abstracts Service, *C* carbon, *Cl* chlorine, *H* hydrogen, *K* potassium, *Mg* magnesium, *N* nitrogen, *Na* sodium, *O* oxygen, *P* phosphorus

Plastic drainage pipes were used for wetland construction (Supplementary Material 1). All 18 wetlands had heights of 100 cm and diameters of 10 cm. All wetlands were filled to 90 cm with washed gravel, applying two layers of aggregates. Large gravel (diameter; 10–20 mm) was used at the bottom, preventing clogging. Pea gravel (diameter; 5–10 mm) was at the top of each wetland. The outlet valves were at the centre of the bottom plate of each filter.

All wetlands contained *Phragmites australis*, which was monitored for health and growth. Dead plants were cut to about 13 cm in terms of height. The corresponding cuttings were recycled within the filters.

The aquatic fertiliser TNC Complete was purchased from TNC Limited (Spotland Bridge Mill, Mellor Street, Rochdale, UK) and applied in the experimental research as a nutrient for the plants and microorganisms. The associated key ingredients were phosphorus (0.2%), nitrogen (1.5%), iron (0.08%), manganese (0.018%), potassium (5%), magnesium (0.08%), copper (0.002%), molybdenum (0.001%), boron (0.01%) and zinc (0.01%). TNC Complete also provides ethylenediaminetetraacetic acid (EDTA) that is a source of the elements copper, iron, manganese and zinc. One millilitre of fertiliser was added to 10 l of tap water.

The packing order of the experimental constructed wetland set-up treating artificial wastewater containing two azo textile dyes is shown in Table 3. All wetlands were filled with the same washed gravel.

**Table 3:** Packing order of the experimental constructed wetland set-up treating artificial wastewater containing two azo textile dyes

Wetland number	Plants	Dye			Resting time (h)	Contact time (h)
		Type	Mean (mg/l)	SD		
1	No	BR46	6.15	0.75	2	94
2	No	AB113	7.50	1.66	2	94
3	Yes	AB113	7.50	1.66	2	94
4	Yes	Mix	0.153		2	94
5	Yes	BR46	6.15	0.75	2	94
6	Yes	Mix	0.153		2	94
7	Yes	BR46	6.15	0.75	48	48
8	Yes	Mix	0.153		48	48
9	Yes	AB113	7.50	1.66	48	48
10	Yes	Mix	0.153		48	48
11	Yes	BR46	206	9.60	48	48
12	Yes	Mix	5.331		48	48
13	Yes	AB113	207	13.70	48	48
14	Yes	Mix	5.331		48	48
15	Yes	BR46	206	9.60	96	96
16	Yes	Mix	5.331		96	96
17	Yes	AB113	207	13.70	96	96
18	Yes	Mix	5.331		96	96

Mix, mixture between BR46 and AB113, and the reading is in a wavelength

*SD* standard deviation, *BR* basic red, *AB* acid blue

### Analytical methods and equipment

#### Measurement of physical parameters

The physical parameters included dye concentration, colour, total suspended solids (TSS), dissolved oxygen (DO), turbidity, pH, redox potential, electric conductivity (EC) and temperature. Dye concentration, colour and TSS were measured by the spectrophotometer Hach Lange DR2800 (Pacific Way, Salford, UK). Dye concentrations were quantified through a selective wavelength at maximum absorbance for each dye. Colour was measured using a unit Pt/Co scale. The TSS were measured in milligrams/litre. Samples were filtered by using Whatman grade 1 qualitative filter paper (standard grade; circle, 320 mm), which was bought from the Scientific Laboratory Supplies (Wilford Industrial Estate, Wilford, Nottingham, UK).

The DO was estimated using a Hach Lange HQ30D Flexi Meter (Pacific Way, Salford, UK) promptly after taking samples. Turbidity (NTU) was measured by using a TurbiCheck Portable Turbidity Meter (Lovibond Water Testing, Tintometer Group, Division Street, Chicago, IL, USA). The pH (−) and redox potential (mV) were determined by applying a portable WTW VARIO pH meter (Wissenschaftlich-Technische Werkstätten, Weilheim, Germany). The equipment was calibrated with standardised buffer solutions of pH 4, 7 and 9, whenever required. The acceptable range of pH is from 6.5 to 9 (Boyd and Gautier 2000).

The EC (μS/cm) was determined applying a portable Mettler Toledo Education Line Conductivity Meter (Boston Road, Leicester, UK). Although EC itself is not of aquatic or human health concern, its value gives an indication, if there is any other water quality problem. A sudden increase in EC values indicates that there is a source of dissolved ions in the wetland filter (Kumar and Chopra 2012). Furthermore, the site temperature was noted each day, applying a thermometer which was located alongside the wetland filters.

#### Measurement of chemical parameters

The chemical parameters included COD, ammonia nitrogen (NH_4_-N), nitrate nitrogen (NO_3_-N), PO_4_-P and amines. The spectrophotometer Hach Lange DR2800 was applied for the water quality analysis for parameters such as COD, PO_4_-P, NO_3_-N and ammonium nitrogen (NH_4_-N) with milligrams per litre (mg/l) as a unit. The aromatic amines were measured as absorbance by using a WPA Biowave II UV/visible spectrophotometers (Cambourne, Cambridge, UK). Specific wavelengths for the absorbance of every type of aromatic amine exist. Also, samples were filtered by using a specific filter paper (Whatman grade 1 qualitative filter paper, standard grade, circle, 320 mm). The water quality analysis was performed according to APHA (1995), if not clarified otherwise. Liquid samples were taken between 10:00 and 11:00 a.m.

The Shapiro-Wilk test (Shapiro and Wilk 1965; Razali and Wah 2011) was applied to judge data normality. A one-way analysis of variance (ANOVA) test was performed with the help of the Statistical Package for the Social Sciences software to analyse normally distributed data. The Mann-Whitney test was applied to evaluate non-normal data (Stoline 1981; Kasuya 2001). The ANOVA and Mann-Whitney tests compared averages between various treatments (e.g. Table 4).

**Table 4:** Application of the statistical wetland filter set-up design (Table 3) to assess the impact of individual key variables

Comparison of two wetland systems with each other		Impact to be assessed
First wetland with number	Second wetland with number	
1	2	Difference between BR46 and AB113
1	5	*Phragmites australis* on BR46
2	3	*Phragmites australis* on AB113
4	6	Mixing dyes (low concentration)
5	7	Decrease in contact time (or increase in resting time on BR46)
3	9	Decrease in contact time (or increase in resting time on AB113)
7	9	Difference between BR46 and AB113
8	10	Mixing dyes (low concentration)
7	11	Increased BR46 concentration
9	13	Increased AB113 concentration
12	14	Mixing dyes (high concentration)
11	15	Increased contact and resting times
13	17	Increased contact and resting times
11	13	Difference between BR46 and AB113
15	17	Difference between BR46 and AB113
16	18	Mixing dyes (high concentration)

*BR* basic red, *AB* acid blue

## Results and discussion

### Test of normality for plant and liquid samples

Test of normality findings concerning the dimensions of *P. australis* and effluent water quality variables is shown in Supplementary Material S3.

### Plant growth assessment

Plants became yellow in winter. Dead plant parts were cut and recycled within the wetlands (Stefanakis et al. 2014). Plants subjected to the dye AB113 developed well compared to those linked to BR46. Plants for systems with long contact time grew better than those plants associated with short time (Table 5). These findings support similar ones by Pagter et al. (2005).

**Table 5:** Dimensions of *Phragmites australis* (Cav.) Trin. ex Steud. (common reed) planted in the experimental wetlands

Dye	Wetland number	Number of stems	Characteristics					
			Length (cm)			Diameter (mm)		
			Minimum	Maximum	Mean ± SD	Minimum	Maximum	Mean ± SD
BR46	5	68	69	142	108 ± 17.8	1.1	3.1	2.2 ± 0.58
	7	20	60	122	93 ± 16.9	0.8	2.3	1.1 ± 0.39
	11	17	45	90	67 ± 11.2	0.8	1.1	0.9 ± 0.08
	15	14	44	90	60 ± 12.9	0.8	1.4	1.0 ± 0.16
AB113	3	40	79	140	107 ± 18.0	1.0	3.4	2.1 ± 0.63
	9	15	55	98	79 ± 14.8	0.8	1.2	1.0 ± 0.11
	13	20	45	98	70 ± 16.3	0.7	2.9	1.9 ± 0.59
	17	10	29	56	41 ± 9.1	0.7	1.6	1.1 ± 0.26
Mixture of BR46 and AB113	4	48	78	142	109 ± 15.9	1.0	3.9	2.4 ± 0.73
	6	55	70	134	109 ± 16.2	1.1	3.7	2.3 ± 0.59
	8	7	85	101	94 ± 5.5	0.9	2.0	1.5 ± 0.39
	10	10	80	110	95 ± 8.2	1.1	2.3	1.8 ± 0.37
	12	10	45	87	64 ± 11.3	0.8	1.6	1.0 ± 0.22
	14	16	46	80	66 ± 9.7	1.3	3.1	2.1 ± 0.48
	16	7	45	61	54 ± 5.8	0.8	1.9	1.3 ± 0.37
	18	9	44	67	58 ± 7.5	1.0	2.9	1.8 ± 0.63

*BR* basic red, *AB* acid blue, *SD* standard deviation

Regarding plant growth, there was a significant (*ρ* < 0.05) difference concerning the length and diameter at low and high AB113 concentrations (wetlands 3, 9, 13 and 17). Concerning plant growth at the presence of BR46, significant (*p* < 0.05) differences for the length and diameter at the low dye concentrations were recorded (wetlands 5 and 7). No significance (*p* > 0.05) for either parameter was noted for the high dye concentrations (wetlands 11 and 15). In case of the mixed dye, there was no significant (*p* > 0.05) difference regarding the length at the low and high dye concentrations (wetlands 4, 6, 8, 10, 12, 14, 16 and 18). While with respect to the plant diameter, there was no significant (*p* > 0.05) difference for wetlands 12 and 14 (high concentration).

### Redox potential and dissolved oxygen

Redox potentials above 100 mV are linked to aerobic environments. In comparison, values below − 100 mV highlight anaerobic boundary conditions (Suthersan 2001). The DO is an important parameter in constructed wetlands, since it is essential for aerobic respiration for microorganisms and it regulates the oxidation-redox potential in wastewater (Boyd 2000). Wu et al. (2011b) and Hou et al. (2016) highlighted that the main pathways for oxygen transfer in constructed wetlands such as the system in this research (tidal flow) are wetland macrophytes releasing oxygen via their roots, contact transfer at the interface of biofilm and atmosphere and DO associated with influent wastewater. In case of low concentration, redox potential values (Table 6) for the effluent of BR46, AB113 and the mixture of both of them were in the range between − 34 and − 64 mV, and for the effluent high concentrations, the values were in the range between − 56 and − 95 mV. These results show dye degradation, regardless of aerobic and anaerobic conditions. Regarding DO for both dyes (BR46 and AB113), the lowest effluent values (Table 6) were noted for planted wetlands 5 and 3 (low resting and high contact times of 2.97 and 3.37 mg/l, respectively) when compared with the unplanted wetlands 1 and 2 and planted wetlands 7 and 9 (high resting time and low contact time), respectively. Concerning the mixture between the two dyes, the value of DO for wetlands 4 and 6 (low resting and high contact times) was lower than that for wetlands 8 and 10 (high resting and low contact times) as a result of the higher contact time leading to consumption of more DO by the microbial community. The same findings for the DO between wetlands 11 and 13 and wetland 12 (low resting and contact times) and between wetlands 15 and 17 and wetland 16 (high resting and contact times) concerning a high concentration for BR46, AB113 and the mixture between them were noted. The result was opposite between wetlands 14 (low resting time and low contact time) and 18 (high resting time and high contact time). Furthermore, in the case of low and high concentrations of the two dyes and the mixture of both of them during spring time, wetlands with higher resting time started to consume more DO when compared to wetlands with lower resting time, because the increase in aerobic microorganisms was greater than that of the anaerobic ones.

**Table 6:** Inflow and outflow water quality characteristics for general physical and chemical variables related to different wetlands

Dye	Type of flow	Wetland number	No. of samples	Characteristics																	
				pH			Redox potential (mV)			Dissolved oxygen (mg/l)			Total suspended solids (mg/l)			Turbidity (NTU)			Electric conductivity (μS/cm)		
				Min.	Max.	Mean ± SD	Min.	Max.	Mean ± SD	Min.	Max.	Mean ± SD	Min.	Max.	Mean ± SD	Min.	Max.	Mean ± SD	Min.	Max.	Mean ± SD
BR46	In	N/A	82	7.22	7.77	7.47 ± 0.17	− 56	− 41	− 47.72 ± 3.74	8.64	9.66	9.24 ± 0.22	1	2	1.07 ± 0.26	2.01	6.13	3.91 ± 0.85	545	575	560 ± 9.4
	Out	1	82	7.37	8.14	7.72 ± 0.23	− 67	− 49	− 55.98 ± 3.81	2.13	5.51	3.73 ± 0.92	0	8	4.48 ± 1.56	3.43	6.61	5.24 ± 0.69	451	558	483 ± 18.0
	Out	5	82	6.94	7.36	7.17 ± 0.08	− 45	− 26	− 35.29 ± 4.31	2.01	4.06	2.97 ± 0.46	2	45	10.68 ± 9.52	4.11	76.5	11.81 ± 17.02	487	670	644 ± 35.8
	Out	7	82	7.08	8.32	7.57 ± 0.42	− 51	− 30	− 41.80 ± 4.74	2.38	5.21	3.71 ± 0.66	0	6	2.73 ± 1.45	3	6.53	4.07 ± 0.77	336	421	397 ± 16.6
	In	N/A	81	6.79	7.25	6.94 ± 0.14	− 24	− 14	− 19.43 ± 2.48	8.8	9.74	9.21 ± 0.18	35	56	45.01 ± 4.51	12.6	15.9	14.62 ± 0.63	685	765	735 ± 13.5
	Out	11	81	7.16	8.01	7.49 ± 0.24	− 97	− 38	− 56.28 ± 14.03	2.58	4.73	3.65 ± 0.46	16	46	23.68 ± 5.67	7.23	27.1	13.11 ± 3.43	563	684	604 ± 16.1
	Out	15	41	7.81	8.21	7.98 ± 0.09	− 87	− 67	− 72.90 ± 4.22	2.41	4.01	3.03 ± 0.36	11	32	14.95 ± 3.19	8.04	23.4	12.09 ± 3.94	559	644	581 ± 16.6
AB113	In	N/A	82	7.19	7.57	7.35 ± 0.08	− 59	− 39	− 44.85 ± 3.21	8.41	9.89	9.40 ± 0.35	4	8	5.87 ± 1.04	5.06	6.71	7.51 ± 0.42	521	575	543 ± 12.9
	Out	2	82	7.37	7.72	7.59 ± 0.07	− 66	− 50	− 60.71 ± 3.08	2.42	5.57	4.14 ± 0.78	3	12	7.96 ± 1.72	3.91	6.99	5.37 ± 0.87	468	601	559 ± 22.5
	Out	3	82	7.21	7.77	7.57 ± 0.16	− 71	− 46	− 64.24 ± 4.90	2.62	4.76	3.37 ± 0.54	2	22	6.71 ± 4.04	4.86	10.49	7.03 ± 1.18	497	622	583 ± 21.5
	Out	9	82	7.26	7.71	7.51 ± 0.07	− 63	− 44	− 57.90 ± 3.42	2.36	5.45	3.59 ± 0.79	0	7	3.16 ± 1.28	2.87	6.42	4.59 ± 0.78	408	511	484 ± 16.4
	In	N/A	81	7.99	8.17	8.07 ± 0.04	− 90	− 75	− 82.12 ± 4.27	9.00	9.51	9.31 ± 0.11	97	126	110 ± 8.59	42.31	62.34	51.79 ± 3.95	855	892	870 ± 5.6
	Out	13	81	7.83	8.29	8.16 ± 0.12	− 102	− 74	− 92.24 ± 6.78	1.95	5.21	4.07 ± 0.61	37	78	62.64 ± 8.47	9.95	34.21	24.21 ± 6.61	702	909	765 ± 51.3
	Out	17	41	7.80	8.31	8.15 ± 0.16	− 106	− 78	− 95.27 ± 8.27	1.98	4.41	3.62 ± 0.73	40	97	58.29 ± 14.40	12.45	25.41	18.62 ± 4.24	672	899	742 ± 60.8
The mixture of the dyes	In	N/A	82	7.2	7.39	7.32 ± 0.03	− 52	− 35	− 45.67 ± 3.01	8.78	9.62	9.36 ± 0.19	6	10	8.32 ± 0.89	4.23	5.92	5.14 ± 0.34	510	535	522 ± 6.2
	Out	4	82	7.01	7.49	7.29 ± 0.09	− 46	− 28	− 40.62 ± 3.02	1.67	4.47	3.12 ± 0.59	7	62	12.99 ± 10.90	3.85	64.5	10.67 ± 10.95	478	586	508 ± 14.2
	Out	6	82	6.78	7.18	7.09 ± 0.07	− 39	− 17	− 33.61 ± 3.06	2.21	4.01	2.98 ± 0.44	1	72	11.40 ± 17.02	4.14	87.4	11.67 ± 17.43	475	568	491 ± 13.0
	Out	8	82	7.21	7.51	7.33 ± 0.07	− 59	− 41	− 50.80 ± 3.81	2.01	5.01	3.85 ± 0.63	0	6	2.95 ± 1.22	3.54	6.66	4.41 ± 0.55	339	460	399 ± 18.6
	Out	10	82	7.01	7.51	7.27 ± 0.14	− 55	− 30	− 46.66 ± 5.25	1.89	4.84	3.55 ± 0.68	0	6	3.07 ± 1.38	3.42	6.93	4.65 ± 0.61	335	439	413 ± 24.2
	In	N/A	81	7.2	7.46	7.35 ± 0.04	− 47	− 26	− 34.91 ± 5.16	8.84	9.72	9.24 ± 0.15	242	294	263 ± 16.54	131	157	144 ± 5.97	760	785	775 ± 4.9
	Out	12	81	7.71	7.9	7.78 ± 0.03	− 81	− 60	− 72.64 ± 4.67	2.7	5.05	4.29 ± 0.62	67	239	100 ± 44.63	14.2	71.3	43.66 ± 18.77	678	734	710 ± 9.8
	Out	14	81	6.35	7.75	7.60 ± 0.15	− 72	− 48	− 61.23 ± 4.68	2.19	4.52	2.98 ± 0.63	57	128	80.48 ± 11.56	11.3	55	31.87 ± 11.95	693	760	738 ± 9.4
	Out	16	41	7.75	8.01	7.86 ± 0.06	− 86	− 65	− 75.32 ± 4.54	1.56	5.19	3.52 ± 0.87	67	252	109 ± 46.20	20.4	68.3	44.59 ± 13.99	670	732	717 ± 11.0
	Out	18	41	7.74	8.17	8.00 ± 0.08	− 89	− 71	− 82.07 ± 4.58	1.42	5.50	3.86 ± 1.06	42	112	75.27 ± 18.37	14.9	51.2	32.64 ± 10.31	696	785	746 ± 13.3

*BR* basic red, *AB* acid blue, *SD* standard deviation, *Min.* minimum, *Max.* maximum, *N/A* not applicable

### Conductivity, suspended solids and turbidity

The EC is commonly applied as an indicator for ion-carrying species (Islam et al. 2011), and corresponding EC values may be used as an indicator for other water quality challenges. Any sudden increase in EC value indicates that there is a source of dissolved ions in the wetland filter (Kumar and Chopra 2012). In comparison, all effluent values for all wetlands in cases of low and high concentrations for both dyes and the dye mixture were compliant with the national effluent discharge quality standards set by the Government of Bangladesh, which stated that the maximum effluent of EC for inland surface water, public sewer secondary treatment plants and irrigated land is 1200 μS/cm (Ahmed et al. 2002). Furthermore, the Sri Lanka Central Environmental Authority (2008) stated that the maximum EC discharge on land for irrigation purpose is 2250 μS/cm. Reference to standards set on the Indian sub-continent is made here, because corresponding countries produce most of the dye wastewater being discharged to the environment.

Concerning the low concentration of BR46 and AB113 (Table 6), a higher elevation was found in planted wetlands 5 and 3 (contact time 94 h), respectively, when compared to the unplanted control wetlands 1 and 2 (contact time 94 h), respectively, while a decrease in EC effluent values was found in wetlands 7 and 9 (contact time 48 h), respectively. For the high concentration for both dyes (BR46 and AB113), the EC effluent values for all wetlands were less than the influent values. Furthermore, wetlands 15 and 17 (long resting and contact times) had EC values less than wetlands 11 and 13 (low resting time and low contact time), respectively. Regarding the dye mixture for both low and high concentrations, all effluent values were less than the influent ones as shown in Table 6. Nevertheless, all previous results indicated no sudden increase in EC values for all wetlands.

The measurement of the conventional pollutant TSS is essential for water treatment works design (Dzurik 2003; Bell et al. 2011). Concerning low concentrations of BR46 and AB113, there were increases in TSS effluent for all wetlands when compared to the influent as shown in Table 6. A lower increase was found in the planted wetlands 7 and 9 (high resting and low contact times) when compared with the unplanted wetlands 1 and 2 and the planted wetlands 5 and 3 (low resting and high contact times), respectively. For the mixture of both dyes, a slight increase of TSS was found for wetland 4, while a decrease was recorded for wetlands 6, 8 and 10. In case of high concentrations for both dyes (BR46 and AB113) and the mixture of the two dyes, a good TSS reduction was recorded for all wetlands as shown in Table 6. Wetlands with high resting and contact times had a lower TSS effluent concentrations, when compared with wetlands, which have low resting and contact times.

All wetland effluents of low and high concentrations of BR46, AB113 and the mixture of both dyes (Table 6) were compliant with the national effluent discharge quality standards set by the Government of Bangladesh, which stated that the maximum TSS effluent concentrations for inland surface water, public sewer secondary treatment plant outflow and irrigated land application are 150, 500 and 200 mg/l, respectively (Ahmed et al. 2002).

A high turbidity of surface water may indicate cloudiness due to elevated concentrations of TSS (Postolache et al. 2007). A higher turbidity value can also increase the temperature of surface water as a result of increased absorption of heat from sunlight, as well as leading to reduced light penetration, which affects photosynthesis (Håkanson 2006).

For the low concentration of the dye BR46, there was an increase in all effluent wetlands when compared with the influents. The planted wetland 7 (high resting and low contact times) has a smaller increase when compared with the unplanted wetland 1 and the planted wetland 5 (low resting and high contact times). In case of dye AB113, a slight increase was recorded for the mean value of the planted wetland 3 (low resting time and high contact time), while a slight decrease was noted for the unplanted wetland 2 (low resting and high contact times) and the planted wetland 9 (high resting time and low contact time). For the mixture of the two dyes, an increase was recorded in wetlands 4 and 6 (low resting time and high contact time), while a decrease was noted in wetlands 8 and 10 (high resting time and low contact time). Regarding the high concentrations of BR46, AB113 and the mixture of these two dyes, all wetlands had a good effluent reduction when compared with the influent. Wetlands 15, 17, 16 and 18 (high resting and contact times) had a greater reduction when compared with wetlands 11, 13, 12 and 14 (low resting and contact times), respectively.

Lin et al. (2005) and Bulc and Ojstršek (2008) stated that the ability of vertical-flow constructed wetlands to reduce TSS and turbidity is relatively poor. In this study, for a low concentration of AB113, a short contact time (48 h) was more advantageous than a long (94 h) one for the reduction of TSS and turbidity as well as in the case of the mixture of both of the dyes (BR46 and AB113). While for high concentrations of both dyes and a mixture of both dyes, the long contact time was better than the short contact time. The percentage TSS reduction rates for BR46, AB113 and the mixture of these dyes were 69, 47 and 71%, respectively.

### pH value

The measuring of pH is very important due to its impact on nutrients, COD and TSS in constructed wetlands. The pH value influences microbial populations in degrading pollutants (Eke and Scholz 2008; Lavrova and Koumanova 2013; Paing et al. 2015). Concerning the low BR46 concentration, the mean influent pH was 7.47, a minute decrease in the pH effluent value of 0.3 was noted in the planted wetland 5 (low resting time and high contact time), while there was a slight increase of 0.25 and 0.1 for the unplanted wetland 1 (low resting time and high contact time) and the planted wetland 7 (high resting and low contact times), respectively. For the dye AB113, there was a slight effluent increase of 0.24, 0.22 and 0.16 for the unplanted control wetland 2, planted wetland 3 (low resting and high contact times) and planted wetland 9 (high resting and low contact times), respectively, when compared to the influent value of 7.35 as shown in Table 6. In case of the mixture of both dyes, there was a slight decrease of 0.03, 0.23 and 0.05 for wetland 4, wetland 6 (low resting and high contact times) and wetland 10 (high resting and low contact times), respectively, if compared with the influent value of 7.32, while for wetland 8 (high resting and low contact times), there was a slight increase of 0.01. For the high concentration for both dyes (BR46 and AB113) and the mixture of the two dyes, a slight increase was found ranging between 0.08 and 0.65 for wetlands 11, 13, 12, 14 (low resting and contact times), 17, 16 and 18 (high resting and contact times), while an increase of 1.04 was recorded for wetland 15 (high resting and contact times), when compared with the corresponding influent value of 6.94. This increase in effluent pH values is due to the formation of basic aromatic amine metabolites (Chandra 2015).

Regarding the effect of plants on the pH value for the low concentration of the dye AB113, there was a slight difference of 0.02 between the unplanted control wetland 2 and the planted wetland 3 (both of them have the same conditions). This result suggests that the pH modification in vertical-flow constructed wetlands is probably as a result of interactions between the media and its biofilms, rather than due to the plants; this result confirms findings by Kadlec and Wallace (2008). Unlike the result for the dye BR46, there was a difference of 0.55 between the unplanted control wetland 1 and the planted wetland 5 (both of them have the same conditions). The different results regarding the role of plants on pH are most likely due to each dye having a different chemical structure and molecular weight as shown in Table 3. Furthermore, there were no change in pH values in contrast to the findings, which were obtained by Wieder (1989), who surveyed 128 constructed wetlands treating acid coal mine wastewater and found a difference of 0.11(influent pH was 2.5) between effluent and influent. Mitsch and Wise (1998) corroborated this finding; they found that the difference between the influent and the effluent is 0.52 (influent pH was 2.82).

Kadlec and Wallace (2008) stated that the pH value for most bacteria responsible for degradation is between 4 and 9.5. Nevertheless, findings indicate the ability of macrophytes to modify pH conditions in the rhizosphere (Brix et al. 2002). Furthermore, the effluent pH values for all wetlands in case of low and high concentrations of BR46, AB113 and the mixture of these dyes during the whole period were compared with the effluent discharge quality standards set by the Government of Bangladesh and the Sweden Textile Water Initiative, which state that the pH effluent for inland surface waters, public sewer secondary treatment plants and irrigated land should be between 6 and 9 (Ahmed et al. 2002; STWI 2012).

### Dye, colour and chemical oxygen demand reductions

The degradation of azo dyes in aerobic and anaerobic environments involves enzymes and chemical reduction (Khehra et al. 2005; Pandey et al. 2007; Saratale et al. 2011). The first contaminant to be easily recognised in an effluent textile wastewater is colour, which adsorbs and reflects sunlight entering the water, thereby interfering with the aquatic species growth and hindering photosynthesis (Pereira and Alves 2012; Yadav et al. 2012).

**Fig. 1:**
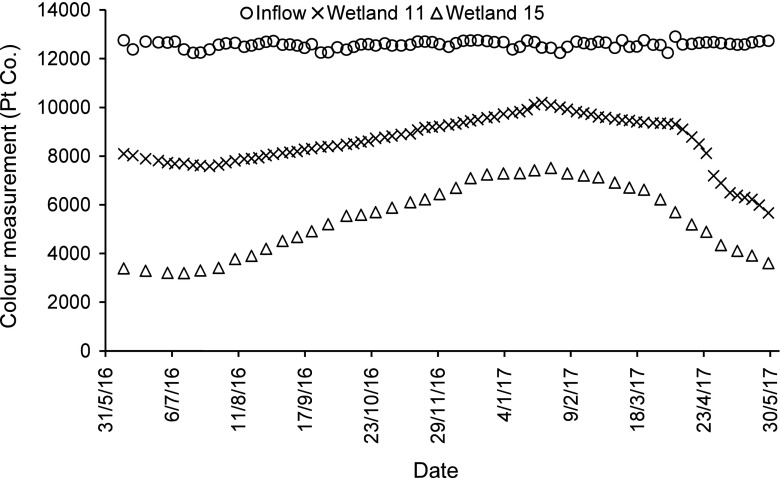
Inflow and outflow colour measurements of Basic Red 46 for wetlands 11 and 15

**Fig. 2:**
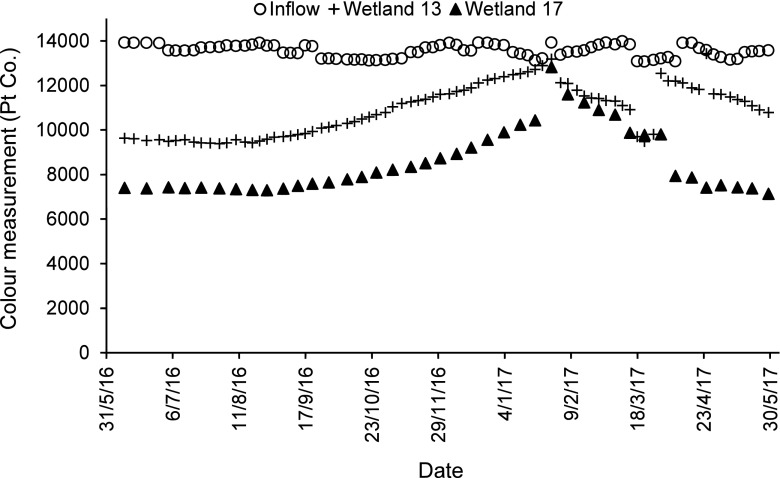
Inflow and outflow colour measurements of Acid Blue 113 for wetlands 13 and 17

**Fig. 3:**
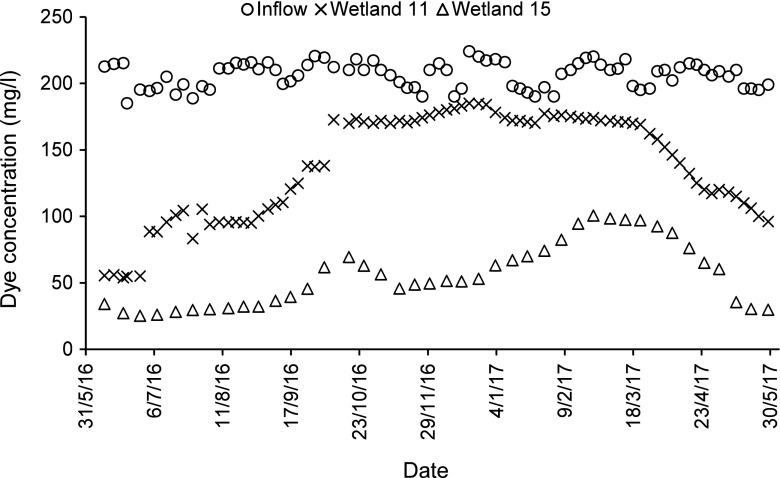
Inflow and outflow dye concentrations of Basic Red 46 for wetlands 11 and 15

**Fig. 4:**
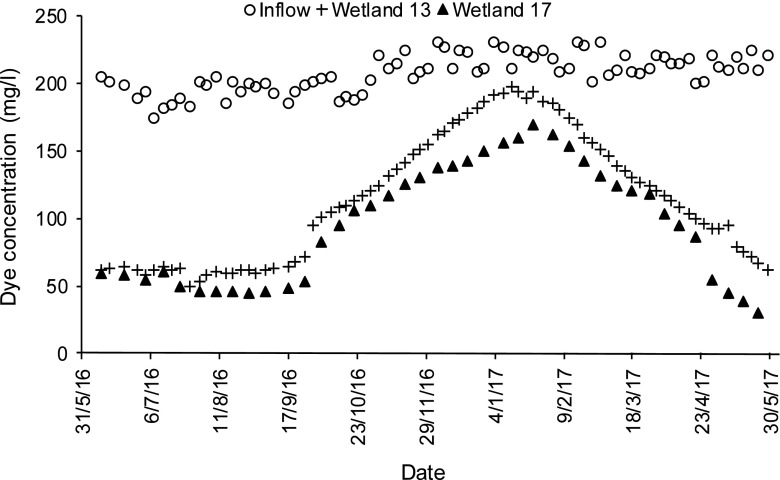
Inflow and outflow dye concentrations of Acid Blue 113 for wetlands 13 and 17

For dye and colour reductions concerning low concentrations of dyes (BR46 and AB113), and the mixture of these two dyes, wetlands with long contact times have the best dye and colour reductions (regardless of the planting regime), when compared to wetlands having short contact times. For the high concentration of the dyes BR46 and AB113 (Figs. 1, 2, 3 and 4), and the mixture of both of them, wetlands, which have a low loading rate (high resting and contact times), have better dye and colour reductions (*p* < 0.05), if compared to wetlands with a high loading rate (low resting and contact times) as shown in Table 7, although wetlands that have a low loading rate have better dye reductions when compared with wetlands which have a high loading rate. The influent values expressed as a mass loading rate for wetlands 11 and 13 (high loading rate) were 573.71 ± 26.74 and 576.49 ± 38.15 g/m^2^/day, respectively, while for wetlands 15 and 17 (low loading rate), they were 286.86 ± 13.37 and 288.25 ± 19.08 g/m^2^/day, respectively (Table 8). The final decision about which loading rate (low or high) is better for a treatment system depends on the design conditions of the specific constructed wetland in the field. The effluent colour values for those wetlands of low concentrations concerning BR46, AB113 and the corresponding mixture of these dyes were compliant with the national effluent discharge quality standards set by the Government of India (1986), which stated the maximum colour value is 400 Pt/Co. In case of the high concentrations for BR46, AB113 and the mixture of these two dyes, they were not compliant even when compared to the maximum threshold for colour (550 Pt/Co.) set by the Government of Taiwan (2003).

**Table 7:** Colour, dye and chemical oxygen demand (COD) reduction for different wetlands (Tables 3 and 4)

Dye	Flow type	Wetland number	No. of samples	Characteristics											
				Colour (Pt/Co)				Dye concentration (mg/l)				COD^a^			
				Min.	Max.	Mean ± SD	Reduction (%)	Min.	Max.	Mean ± SD	Reduction (%)	Min.	Max.	Mean ± SD	Reduction (%)
BR46	In	N/A	82	410	439	422 ± 7.7	N/A	4.3	8.4	6.2 ± 0.75	N/A	200	297	248 ± 20.9	N/A
	Out	1	82	16	187	71 ± 35.3	83	0.0	1.2	0.5 ± 0.22	92	50	93	76 ± 11.8	69
	Out	5	82	26	356	97 ± 71.4	77	0.0	1.0	0.6 ± 0.24	91	54	110	74 ± 16.4	70
	Out	7	82	9	272	120 ± 80.5	72	0.1	1.9	0.7 ± 0.40	89	20	80	45 ± 13.9	82
	In	N/A	81	12,240	12,900	12,574 ± 142.2	N/A	185.0	224.0	206.0 ± 9.60	N/A	478	576	511 ± 36.7	N/A
	Out	11	81	5670	10,210	8604 ± 1053.0	32	53.8	185.0	139.0 ± 39.18	33	196	385	311 ± 52.4	39
	Out	15	41	3200	7520	5442 ± 1459.5	57	25.1	100.6	55.8 ± 24.00	73	135	301	225 ± 48.4	56
AB113	In	N/A	82	530	589	555 ± 16.1	N/A	5.3	10.9	7.5 ± 1.66	N/A	234	310	275 ± 18.1	N/A
	Out	2	82	39	386	135 ± 100.3	76	0.5	4.8	1.2 ± 0.87	85	54	125	66 ± 16.3	76
	Out	3	82	89	400	182 ± 82.5	67	0.6	4.5	1.4 ± 0.84	82	89	176	106 ± 19.4	62
	Out	9	82	224	490	310 ± 71.2	44	0.6	5.3	1.7 ± 1.08	77	40	85	53 ± 10.3	81
	In	N/A	81	13,080	13,990	13,561 ± 279.7	N/A	174.0	231.0	207.0 ± 13.70	N/A	541	710	599 ± 47.7	N/A
	Out	13	81	7230	13,420	10,699 ± 1338.9	21	50.0	197.0	115.0 ± 46.97	44	199	495	357 ± 75.5	40
	Out	17	41	7290	12,820	8707 ± 1432.6	36	30.2	169.0	95.8 ± 43.72	54	190	345	265 ± 43.3	56
Mixture of BR46 and AB113^b^	In	N/A	82	323	450	400 ± 20.8	N/A	0.108	0.182	0.154 ± 0.022	N/A	254	350	292 ± 28.0	N/A
	Out	4	82	145	311	192 ± 37.5	52	0.031	0.156	0.069 ± 0.025	55	86	270	133 ± 43.6	55
	Out	6	82	80	263	119 ± 33.8	70	0.014	0.157	0.050 ± 0.029	68	58	276	117 ± 54.4	60
	Out	8	82	114	339	233 ± 34.0	42	0.039	0.136	0.067 ± 0.021	56	36	91	57 ± 15.7	81
	Out	10	82	206	372	273 ± 31.4	32	0.044	0.138	0.073 ± 0.017	53	32	120	63 ± 24.8	79
	In	N/A	81	16,090	16,190	16,130 ± 29.9	N/A	4.652	5.781	5.339 ± 0.310	N/A	480	594	551 ± 43.5	N/A
	Out	12	81	8620	14,520	12,199 ± 1593.5	24	3.040	5.333	4.371 ± 0.606	18	145	450	347 ± 76.4	37
	Out	14	81	10,790	13,720	12,020 ± 668.2	25	1.884	5.073	3.587 ± 0.936	33	215	431	340 ± 64.2	38
	Out	16	41	9490	13,690	11,933 ± 1096.6	26	1.521	4.892	3.429 ± 0.997	36	225	397	310 ± 47.1	44
	Out	18	41	6430	12,260	10,250 ± 1680.3	36	1.100	4.511	2.819 ± 1.032	47	230	373	294 ± 34.6	46

*BR* basic red, *AB* acid blue, *SD* standard deviation, *Min.* minimum, *Max.* maximum, *N/A* not applicable

^a^The number of samples is 30

^b^All dye concentration measurements for the mixture are given as a wavelength

**Table 8:** Dye loading rate

Dye	Flow type	Wetland number	No. of samples	Characteristic			
				Dye loading rate (g/m^2^/day)			
				Min.	Max.	Mean ± SD	Reduction (%)
BR46	In	N/A	82	11.98	23.42	17.13 ± 2.09	N/A
	Out	1	82	0.00	3.29	1.45 ± 0.61	92
	Out	5	82	0.00	2.76	1.59 ± 0.67	91
	Out	7	82	0.31	5.21	1.92 ± 1.11	89
	In	11	81	515.22	623.84	573.71 ± 26.74	N/A
	Out	11	81	149.83	515.23	387.12 ± 109.12	33
	In	15	41	257.61	311.92	286.86 ± 13.37	N/A
	Out	15	41	34.95	140.09	77.63 ± 33.42	73
AB113	In	N/A	82	14.82	30.44	20.89 ± 4.63	N/A
	Out	2	82	1.34	13.39	3.20 ± 2.42	85
	Out	3	82	1.69	12.50	3.82 ± 2.34	82
	Out	9	82	1.59	14.79	4.85 ± 3.01	77
	In	13	81	484.59	643.34	576.49 ± 38.15	N/A
	Out	13	81	139.25	548.65	320.28 ± 130.81	44
	In	17	41	242.29	321.67	288.25 ± 19.08	N/A
	Out	17	41	42.05	235.33	133.43 ± 60.88	54

*BR* basic red, *AB* acid blue, *Min.* minimum, *Max.* maximum, *SD* standard deviation, *N/A* not applicable

For textile wastewater, the measurement of COD is very important to assess organic matter in wetlands. Its reduction processes may be aerobic or anaerobic and are based on filtration, adsorption and microbial metabolism processes (Vymazal et al. 1998; Song et al. 2006; Stefanakis et al. 2014). The effluent COD values for a low concentration of BR46, AB113 and the mixture of both dyes (Table 7) were complaint with the national effluent discharge quality standards set by the Government of Bangladesh, which has set the maximum COD values for inland surface water, public sewer secondary treatment plant outflow and irrigation water to be 200, 400 and 400 mg/l, respectively. In case of high concentrations for BR46, AB113 and a mixture of these dyes for both dyes (Table 7), values were compliant for public sewer secondary treatment and irrigated land (Ahmed et al. 2002). For COD reduction concerning the low concentration of dyes (BR46 and AB113) and the mixture of both of these dyes, the results showed that all wetlands demonstrated good COD reduction as shown in Table 7. Furthermore, wetlands with a long resting time had the best COD reductions, if compared to the control (unplanted wetlands) and/or other wetlands having short resting times. These results indicated that both aerobic and anaerobic environments are acceptable for COD reduction. These findings are supported by the DO values for wetlands as shown in Table 6. Wetlands 7, 9, 8 and 10 have effluent DO values higher than those for wetlands 5, 3, 4 and 6. For the high concentration, COD reductions in wetlands, which have low loading rates (high resting and contact times), were better than for those wetlands with high loading rates (low resting and contact times) in terms of COD concentration (Table 7). However, the influent mass loading rates for wetlands 11 and 13 (high loading rate) were 1423.1 ± 102.27 and 1668.2 ± 132.73 g/m^2^/day, respectively, as shown in Table 9, while for wetlands 15 and 17 (low loading rate), they were 711.6 ± 51.14 and 834.1 ± 66.37 g/m^2^/day. The final decision about which wetland performs better depends on the design conditions of constructed wetlands in the field. All previous findings regarding low and high concentrations for BR46, AB113 and the mixture of these two dyes indicate that having both aerobic and anaerobic conditions will improve the COD reduction (Vymazal et al. 1998; Li et al. 2012; Lehl et al. 2016).

**Table 9:** Chemical oxygen demand (COD) loading rate

Dye	Flow type	Wetland number	No. of samples	Characteristics			
				COD loading rate (g/m^2^/day)			
				Min.	Max.	Mean ± SD	Reduction (%)
BR46	In	N/A	30	557.0	827.1	690.7 ± 58.18	N/A
	Out	1	30	137.9	259.0	212.1 ± 32.84	69
	Out	5	30	150.4	306.4	205.2 ± 45.79	70
	Out	7	30	56.0	221.4	124.3 ± 38.68	82
	In	11	30	1331.2	1604.2	1423.1 ± 102.27	N/A
	Out	11	30	545.9	1072.2	866.1 ± 145.85	39
	In	15	30	665.6	802.1	711.6 ± 51.14	N/A
	Out	15	30	188.0	419.1	313.3 ± 67.42	56
AB113	In	N/A	30	651.7	863.4	765.9 ± 50.46	N/A
	Out	2	30	150.1	348.1	185.1 ± 45.34	76
	Out	3	30	247.3	490.2	294.5 ± 54.06	62
	Out	9	30	110.6	235.6	148.5 ± 28.69	81
	In	13	30	1506.7	1977.4	1668.2 ± 132.73	N/A
	Out	13	30	554.2	1378.6	994.3 ± 210.21	40
	In	17	30	753.4	988.7	834.1 ± 66.37	N/A
	Out	17	30	264.6	480.4	369.0 ± 60.31	56

*BR* basic red, *AB* acid blue, *Min.* minimum, *Max.* maximum, *SD* standard deviation, *N/A* not applicable

### Seasonal comparison of effluent dye reductions

The overall seasonal comparison of the influent and effluent dye concentrations for all wetlands is shown in Table 10. In case of low concentration for BR46 and AB113, the best and significant (*ρ* < 0.05) reduction percentages were recorded for the spring season as a result of well-established microbial populations, favourable operating conditions achieved over time and plants, as confirmed by many publications (Scholz et al. 2002; Al-Isawi et al. 2015; Scholz 2015). In case of high concentrations of BR46, AB113 and the mixture of both dyes, the best and significant (*ρ* < 0.05) reduction percentages were linked to summer as shown in Table 9 as a result of the higher temperature as confirmed by several researchers, who stated that the best treatment performance occurs during higher temperatures (Song et al. 2006; Sani et al. 2013).

**Table 10:** Seasonal artificial wastewater removal (mg/l) and for the mixture dyes (absorbance)

Dye	Type of flow	Wetland number(s)	Characteristics							
			Summer^a^		Autumn^b^		Winter^c^		Spring^d^	
			Mean ± SD	Removal (%)	Mean ± SD	Removal (%)	Mean ± SD	Removal (%)	Mean ± SD	Removal (%)
BR46	In	N/A	6.26 ± 0.99	N/A	6.07 ± 0.49	N/A	6.22 ± 0.76	N/A	6.06 ± 0.66	N/A
	Out	1	0.56 ± 0.13	91	0.67 ± 0.23	89	0.47 ± 0.22	92	0.34 ± 0.08	94
	Out	5	0.58 ± 0.18	91	0.58 ± 0.29	90	0.54 ± 0.31	91	0.58 ± 0.16	90
	Out	7	1.00 ± 0.39	84	0.69 ± 0.16	88	0.66 ± 0.37	89	0.28 ± 0.27	95
	In	N/A	202.80 ± 9.52	N/A	207.47 ± 9.64	N/A	208.64 ± 11.05	N/A	204.65 ± 7.18	N/A
	Out	11	95.9 ± 11.94	53	168.30 ± 14.18	19	174.70 ± 4.63	16	128.58 ± 22.27	37
	Out	15	30.94 ± 4.41	85	54.18 ± 8.07	74	80.01 ± 16.99	62	63.71 ± 26.82	69
AB113	In	N/A	8.96 ± 1.55	N/A	6.66 ± 1.15	N/A	6.51 ± 1.36	N/A	7.78 ± 1.17	N/A
	Out	2	1.98 ± 1.13	78	0.96 ± 0.65	86	0.70 ± 0.11	89	0.80 ± 0.18	90
	Out	3	2.24 ± 1.14	75	1.18 ± 0.76	82	1.03 ± 0.15	84	0.85 ± 0.18	89
	Out	9	3.10 ± 0.94	65	1.56 ± 0.53	77	1.25 ± 0.32	81	0.69 ± 0.02	91
	In	N/A	192.33 ± 8.14	N/A	207.58 ± 13.31	N/A	208.00 ± 9.24	N/A	213.82 ± 7.04	N/A
	Out	13	60.68 ± 3.90	68	129.81 ± 27.93	37	172.16 ± 21.45	17	96.49 ± 20.34	55
	Out	17	49.81 ± 5.65	74	109.38 ± 26.88	47	146.48 ± 16.07	30	71.29 ± 33.29	67
Mixture of BR46 and AB113 (absorbance)	In	N/A	0.15 ± 0.02	N/A	0.16 ± 0.02	N/A	0.15 ± 0.02	N/A	0.15 ± 0.03	N/A
	Out	4	0.07 ± 0.001	53	0.06 ± 0.01	63	0.05 ± 0.01	67	0.09 ± 0.05	40
	Out	6	0.05 ± 0.01	67	0.04 ± 0.001	75	0.04 ± 0.00	73	0.08 ± 0.05	47
	Out	8	0.07 ± 0.001	53	0.06 ± 0.01	63	0.06 ± 0.00	60	0.09 ± 0.04	40
	Out	10	0.08 ± 0.001	47	0.07 ± 0.01	56	0.06 ± 0.00	60	0.09 ± 0.03	40
	In	N/A	5.35 ± 0.38	N/A	5.24 ± 0.28	N/A	5.34 ± 0.26	N/A	5.44 ± 0.29	N/A
	Out	12	3.60 ± 0.35	33	4.57 ± 0.21	13	4.89 ± 0.32	8	4.57 ± 0.41	22
	Out	14	2.37 ± 0.44	56	3.98 ± 0.38	24	4.51 ± 0.43	16	3.62 ± 0.59	33
	Out	16	2.09 ± 0.53	61	3.75 ± 0.45	28	4.32 ± 0.54	19	3.75 ± 0.58	31
	Out	18	1.57 ± 0.52	71	3.34 ± 0.44	36	3.96 ± 0.56	26	2.52 ± 0.0.42	54
Temperature (°C)	N/A	N/A	22.8	N/A	11.6	N/A	8.6	N/A	20.5	N/A

*BR* basic red, *AB* acid blue, *N/A* not applicable, *SD* standard deviation

^a^From 21 6 2016 to 21 September 2016

^b^From 22 September 2016 to 20 December 2016

^c^From 21 December 2016 to 19 March 2017

^d^From 20 March 2017 to 29 May 2017

### Nutrient reduction

The removal of ortho-phosphate-phosphorous is controlled by chemical and physical adsorption, sedimentation, plant uptake, precipitation and microbial uptake in constructed wetland systems (Brix 1997; Vymazal 2007, 2010; Johari et al. 2016). Moreover, many researchers have reported that the reduction efficiency of phosphorous compounds is generally poor within constructed wetlands (Choudhary et al. 2011; Lavrova and Koumanova 2013; Ge et al. 2016).

For low concentrations in case of AB113 and BR46, the reductions for planted wetlands 3 and 5 (low resting time and high contact time) were significantly (*p* < 0.05) better compared to those for the unplanted control wetlands 2 and 1 (low resting time and high contact time) and the planted wetlands 7 and 9 (high resting time and high contact time; Table 11). In case of the mixture of both dyes (BR46 and AB113), wetlands 4 and 6 (low resting and high contact times) had better reduction percentages when compared with wetlands 8 and 10 (high resting and low contact times), respectively (Table 11). Assessing the high concentrations for BR46, AB113 and the mixture of these dyes, wetlands 15 and 17 and wetlands 16 and 18 (high resting and contact times) had lower PO_4_-P effluent concentrations when compared with wetlands 11 and 13 and wetlands 12 and 14 (low resting and contact times), respectively (Table 8). The previous results for low and high concentrations indicate that the reduction efficiency for PO_4_-P was relatively good, especially for wetlands, regardless of planting regime, with long contact times (and lower resting times).

**Table 11:** Inflow and outflow water quality characteristics for nutrients related to different wetlands (Supplementary Material 1 and Table 2)

Dye	Flow type	Wetland number	No. of samples	Characteristics											
				Ammonia nitrogen (mg/l)				Nitrate-nitrogen (mg/l)				Ortho-phosphate-phosphorus (mg/l)			
				Min.	Max.	Mean ± SD	Removal (%)	Min.	Max.	Mean ± SD	Removal (%)	Min.	Max.	Mean ± SD	Removal (%)
BR46	In	N/A	30	16.9	25.7	21.98 ± 2.43	N/A	21.9	30.2	25.07 ± 2.38	N/A	5.4	7.7	6.36 ± 0.68	N/A
	Out	1	30	11.0	22.1	18.72 ± 2.48	15	0.0	0.5	0.21 ± 0.18	99	2.1	5.9	3.49 ± 0.98	45
	Out	5	30	3.7	19.0	14.41 ± 3.53	34	0.0	0.2	0.02 ± 0.05	100	0.0	3.3	1.76 ± 0.83	72
	Out	7	30	2.6	16.1	6.19 ± 4.78	72	0.0	0.7	0.21 ± 0.20	99	3.4	5.9	4.54 ± 0.69	29
	In	N/A	30	21.3	29.7	26.99 ± 2.41	N/A	28.7	38.1	33.47 ± 1.75	N/A	62.5	68.3	65.78 ± 1.75	N/A
	Out	11	30	12.5	31.4	21.67 ± 4.12	20	4.03	16.6	8.41 ± 3.99	75	33.6	52.4	45.33 ± 6.32	31
	Out	15	30	9.2	26.3	20.29 ± 4.79	25	1.4	13.9	6.23 ± 3.61	81	11.9	35.8	19.09 ± 6.70	71
AB113	In	N/A	30	20.4	24.4	23.36 ± 1.02	N/A	19.9	28.2	23.53 ± 2.19	N/A	7.6	11.8	10.34 ± 1.13	N/A
	Out	2	30	9.9	24.3	19.23 ± 4.16	18	0.0	1.0	0.11 ± 0.23	100	2.3	7.3	4.00 ± 1.62	61
	Out	3	30	13.5	36.2	24.48 ± 7.59	− 5	0.0	1.5	0.55 ± 0.43	98	2.0	6.2	3.91 ± 1.07	62
	Out	9	30	9.1	17.4	13.20 ± 2.25	43	0.0	1.1	0.22 ± 0.31	99	5.1	8.3	6.22 ± 0.94	40
	In	N/A	30	26.9	29.0	28.16 ± 0.68	N/A	30.2	37.6	33.05 ± 2.10	N/A	144.0	158.0	156.00 ± 3.27	N/A
	Out	13	30	13.5	27.7	20.89 ± 4.74	26	3.1	7.3	5.59 ± 0.89	83	8.9	95.4	46.15 ± 28.22	70
	Out	17	30	9.7	24.7	18.60 ± 4.98	34	2.9	6.3	4.57 ± 0.99	86	21.2	67.0	42.29 ± 16.47	73
Mixture of BR46 and AB113	In	N/A	30	27.2	34.2	30.27 ± 1.81	N/A	22.6	29.0	25.37 ± 1.72	N/A	6.0	8.7	7.56 ± 0.81	N/A
	Out	4	30	17.4	35.0	26.01 ± 4.75	14	0.0	0.3	0.08 ± 0.09	100	2.5	8.3	4.47 ± 1.48	41
	Out	6	30	7.2	22.2	15.66 ± 4.39	48	0.0	2.1	0.13 ± 0.39	100	0.4	4.2	2.03 ± 1.02	73
	Out	8	30	5.1	17.0	9.78 ± 3.21	68	0.1	13.9	3.71 ± 4.79	85	4.2	8.2	5.85 ± 1.19	23
	Out	10	30	2.3	17.9	10.75 ± 5.39	65	0.0	2.5	0.39 ± 0.59	99	3.9	8.9	6.14 ± 1.54	19
	In	N/A	30	23.2	33.6	30.75 ± 2.81	N/A	31.2	35.1	33.91 ± 1.03	N/A	112.1	123.0	118.00 ± 2.72	N/A
	Out	12	30	10.7	24.5	20.72 ± 3.68	33	4.3	9.7	6.45 ± 1.44	81	5.2	86.8	58.37 ± 24.80	51
	Out	14	30	9.8	26.4	19.87 ± 3.85	35	3.7	7.5	5.79 ± 1.04	83	4.7	97.2	56.34 ± 26.13	52
	Out	16	30	8.0	25.2	17.41 ± 5.47	43	2.9	6.1	4.89 ± 0.86	86	19.5	74.3	57.31 ± 15.98	51
	Out	18	30	8.59	27.7	16.84 ± 5.25	45	2.4	8.4	4.71 ± 1.23	86	26.3	69.7	50.48 ± 13.16	57

*BR* basic red, *AB* acid blue, *SD* standard deviation, *Min.* minimum, *Max.* maximum, *N/A* not applicable

A typical standard set by environment agencies for PO_4_-P reduction concerning secondary wastewater treatment is 2 mg/l (Royal Commission on Sewage Disposal 1915). Effluent PO_4_ *p* values were complaint to this standard for low concentrations of BR46 (planted wetland 5; 1.76 mg/l). In comparison, a slight increase in case of the low concentration for the mixture of these two dyes (wetland 6; 2.03 mg/l) was noted. However, the value was relatively high in case of low concentration of AB113 (planted wetland 3; 3.91 mg/l). For other wetlands, the effluent values of PO_4_-P were much higher than the standard value of 2 mg/l.

Nitrification and denitrification are the main reduction mechanisms of nitrogen in constructed wetlands, and these mechanisms include a two-step process: ammonium is oxidised to nitrite followed by oxidisation of nitrite to nitrate (nitrification process). The subsequent denitrification process involves the reduction of nitrate to gaseous nitrogen (Schaechter 2009; Kessel et al. 2015; Song et al. 2015; Yang et al. 2016). Regarding NH_4_-N reduction percentages for low concentrations of BR46 and AB113 (Table 11), planted wetlands 7 and 9 (high resting and low contact times) have better reduction percentages when compared with the unplanted control wetlands 1 and 2 as well as the planted wetlands 5 and 3 (low resting and high contact times), respectively. In case of a mixture of both dyes, wetlands 8 and 10 (high resting and low contact times) had better reduction percentages compared to wetlands 4 and 6 (low resting time and high contact time), respectively (Table 11). For the high concentrations of BR46, AB113 and the mixture of both dyes, wetlands 15, 17, 16 and 18 (high resting and contact times) have better reduction percentages when comparing them with wetlands 11, 13, 12 and 14 (low resting and contact times), respectively. The previous results indicate that aeration plays a major function in determining the performance of higher nitrogen reduction. These findings are confirmed by many researchers (Vymazal 2007; Wu et al. 2011a; Fan et al. 2013). The effluent NH_4_-N values for all wetlands in case of low and high concentrations for BR46, AB113 and the mixture of both dyes were compared to the traditional UK standard (Royal Commission on Sewage Disposal 1915), which states that the NH_4_-N outflow from the secondary wastewater should not exceed 50 mg/l. Furthermore, both the Government of India (1986) and the Government of Bangladesh (Ahmed et al. 2002) stated that 50 mg/l is an acceptable outflow threshold to protect surface waters.

Regarding NO_3_-N reduction for low concentrations of BR46, AB113 and the mixture of both dyes (Table 11), the influent NO_3_-N values were in the range 23.53 to 25.37 mg/l. The reduction percentages for all wetlands were in the range between 83 and 100%. For the high concentration of BR46, AB113 and the mixture of both dyes, the influent values were approximately 33.45 mg/l and the reduction percentages for all wetlands were in the range from 75 to 86% (Table 11). The NO_3_-N reduction percentages indicate that vertical-flow constructed wetlands have a good ability to reduce nitrogen in high percentages, especially when there is a source of organic carbon, and both dyes have carbon in their chemical structure (Supplementary Material S1). These findings have been confirmed by Lavrova and Koumanova (2014) as well as Shen et al. (2015).

Furthermore, Lavrova and Koumanova (2013) demonstrated that vertical-flow constructed wetlands can effectively reduce NO_3_-N with and without plants with a sufficient organic carbon source. The effluent NO_3_-N values for all wetlands in case of low and high concentrations for BR46, AB113 and the mixture of both dyes were compared to the traditional UK standard, which states that the NO_3_-N outflow concentration should not exceed 50 mg/l (Royal Commission on Sewage Disposal 1915).

### Aromatic amine reductions

Azo dye decolourisation is achieved under aerobic, anaerobic and anoxic conditions (O’Neill et al. 2000; Sponza and Işik 2002; Van Der Zee 2002; Davies et al. 2006). In anaerobic conditions, the azo bond (N=N) cleaves (cutes), and this process releases aromatic amine, which resists any further anaerobic treatment (Brown and Hamburger 1987; Chung and Stevens 1993). Aromatic amine can be reduced under aerobic treatment (Weber and Wolfe 1987; Pinheiro et al. 2004; Ong et al. 2011). The amine compounds are toxic and negatively impact on some bacteria, leading to insufficient dye degradation (Phugare et al. 2011; Holkar et al. 2014). Each dye has one or more types of aromatic amines (Pielesz et al. 2002; Pinheiro et al. 2004). Wetlands can degrade aromatic amines under aerobic conditions (Mbuligwe 2005; Ong et al. 2010, 2011).

**Fig. 5:**
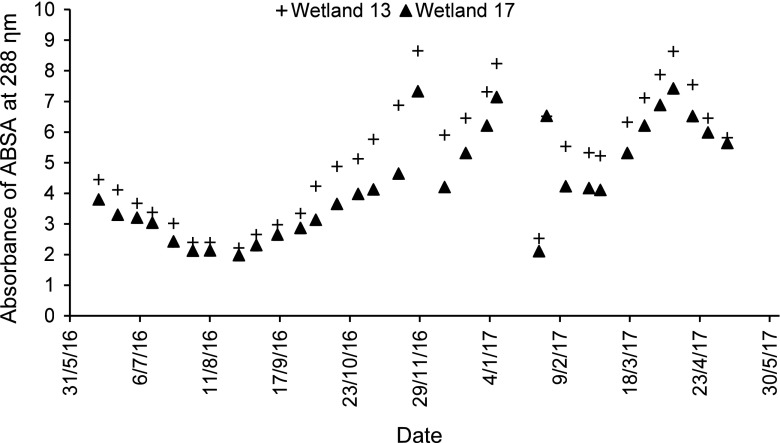
3-Aminobenzenesulfonic acid absorbance for the high concentration of Acid Blue 113

In this study, three types of amines were released as a result of the degradation of the dye AB113: 3-aminobenzenesulfonic acid (ABSA), 1,4-diaminonaphthalene (DAN) and 5-amino-8-(phenylamino)naphthalene-1-sulfonic acid (ANSA) (Senthilvelan et al. 2014). The corresponding wavelengths for maximum absorbance are 288, 255 and 225 nm, respectively (Koepernik and Borsdorf 1983; Paul et al. 1990). In case of BR46, two aromatic amines were released as a result of its degradation: *N*-benzyl-*N*-methylaniline (NBNMA) and *N*-benzyl-*N*-methylbenzene-1,4-diamine (NBNMD), with wavelengths of maximum absorbance of 254 and 290 nm, respectively (Fihtengolts 1969; Küçükgüzel et al. 1999). For the low concentration of AB113, in case of ABSA (Supplementary Material 4), wetland 9 (high resting and low contact times) had a significant (*ρ* < 0.05) reduction efficiency when compared with the unplanted wetland 2 and the planted wetland 3 (low resting time and high contact time). Regarding the high concentration of AB113 and for ABSA amine (Fig. 5), wetland 17 (high resting time and high contact time) has a significant (*ρ* < 0.05) reduction efficiency when compared with wetland 13 (low resting and contact times). It follows that a reduction of aromatic amine compounds requires aerobic conditions (see above). The amines DAN and ANSA were not detected for low and high concentrations by a UV spectrophotometer. This is because both of them are instable, and therefore, they were not detected in solution as confirmed by Davies et al. (2005) and Davies et al. (2006), who also found that using HPLC analysis did not detect this type of amine.

**Fig. 6:**
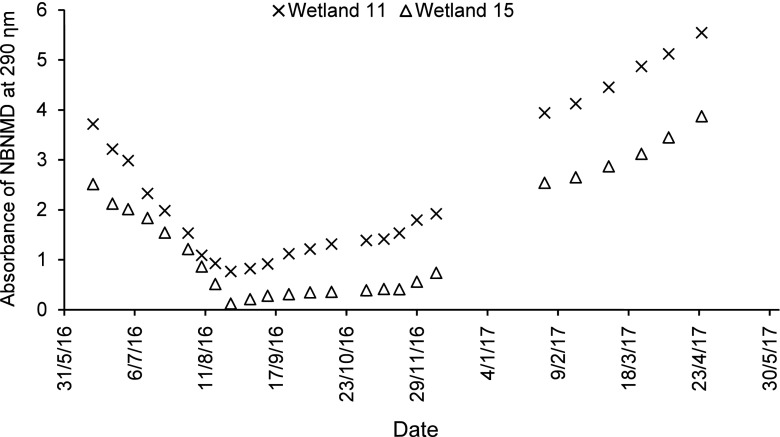
*N*-Benzyl-*N*-methylbenzene-1,4-diamine absorbance for the high concentration of Basic Red 46

For the low concentration of BR46 concerning the NBNMD amine, wetland 7 (high resting and low contact times) has a significant (*ρ* < 0.05) reduction efficiency when compared with the unplanted wetland 1 and the planted wetland 5 (low resting and high contact times) as shown in Supplementary Material 5. The NBNMA amine was not detected by the UV spectrophotometer regarding the unplanted wetland 1, while for the planted wetlands 5 and 7, it was sometimes detected, but this amine was not dependent on the activity of microorganisms required to degrade this type of amine. For the high concentration of BR46 in case of the NBNMD amine (Fig. 6), wetland 15 (high resting and contact times) had a significant (*ρ* < 0.05) reduction efficiency when compared with wetland 11 (low resting and contact times). Furthermore, during the period between 19 December 2016 and 3 February 2017, the NBNMD amine was not detect as a result of a decrease in temperature during this period and because of the growth and development of microbial communities (Jerman et al. 2009). The NBNMA amine was not detected by the UV spectrophotometer for the same reason as stated above.

## Conclusions and recommendations for further research

Regarding low BR46 and AB113 reductions, the unplanted wetlands had good reduction performances, if compared with planted wetlands concerning the removal of dyes. For the high concentrations of AB113, BR46 and a mixture of both of them, wetlands with long contact times were considerably better than wetlands which had short contact times, in terms of dye, colour and COD reductions. For low and high inflow dye concentrations, best removals were recorded for spring and summer in this order. Furthermore, aromatic amine concentrations were very low.

The vertical-flow wetland filters were linked to significantly (*p* < 0.05) good denitrification processes for both low and high concentrations of AB113, BR46 and the mixture of both dyes throughout the year. Regarding nitrate nitrogen (NO_3_-N), the reduction percentage rates of AB113, BR46 and a mixture dye of both of them were between 85 and 100%.

Future wetland designs for the treatment of dye wastewater should be based on these recent more long-term research findings. The authors recommend to assess the effect of pH (low and high) on dye reduction. Aromatic amine compounds require more large-scale process investigations, especially in case of mixtures of dyes.

## Electronic supplementary material

ESM 1 (DOCX 13965 kb)

ESM 2 (DOCX 39 kb)

ESM 3 (DOCX 22 kb)

ESM 4 (DOCX 50 kb)

ESM 5 (DOCX 53 kb)

## Abbreviations

AB: Acid blue
ABSA: 3-Aminobenzenesulfonic acid
ANOVA: Analysis of variance
ANSA: 5-Amino-8-(phenylamino)naphthalene-1-sulfonic acid
AO: Acid orange
AY: Acid yellow
BR: Basic red
CASRN: Chemical Abstracts Service Registry Number
COD: Chemical oxygen demand
DAN: 1,4-Diaminonaphthalene
DO: Dissolved oxygen
DY: Disperse yellow
EC: Electric conductivity
HF: Horizontal-flow
N: Nitrogen
N/A: Not applicable
NBNMA: *N*-Benzyl-*N*-methylaniline
NBNMD: *N*-Benzyl-*N*-methylbenzene-1,4-diamine
NH_4_-N: Ammonia nitrogen
NO_2_-N: Nitrite nitrogen
NO_3_-N: Nitrate nitrogen
PO_4_–P: Ortho-phosphate-phosphorus
RB: Reactive black
SD: Standard deviation
SE: Standard error
TDS: Total dissolved solids
TN: Total nitrogen
TOC: Total organic carbon
TP: Total phosphorus
TSS: Total suspended solids
VF: Vertical-flow
VY: Vat yellow
*λ*_max_: Maximum absorbance
