# Supplementary material for: Treatment of artificial wastewater containing two azo textile dyes by vertical-flow constructed wetlands
Source: Environ Sci Pollut Res Int. 2017 Dec 21;25(7):6870–89. doi: 10.1007/s11356-017-0992-0 (PMC5846842; doi:10.1007/s11356-017-0992-0)
Supplement: Supplementary file 1 — (DOCX 13965 kb) [file 11356_2017_992_MOESM1_ESM.docx]

**Supplementary Material S1**





**Fig. S1** Experimental vertical-flow constructed wetland rig located within a greenhouse at the beginning of the experiment
