# Supplementary material for: Treatment of artificial wastewater containing two azo textile dyes by vertical-flow constructed wetlands
Source: Environ Sci Pollut Res Int. 2017 Dec 21;25(7):6870–89. doi: 10.1007/s11356-017-0992-0 (PMC5846842; doi:10.1007/s11356-017-0992-0)
Supplement: Supplementary file 2 — (DOCX 39 kb) [file 11356_2017_992_MOESM2_ESM.docx]

**Supplementary Material S2**

**Table S2** Details of dyes used in the experimental constructed wetlands (adopted from Hussein and Scholz, 2017)

| Dye | BR46 | AB113 |
| --- | --- | --- |
| Molecular weight | 401.3 | 681.65 |
| Molecular formula | C_18_H_21_BrN_6_ | C_32_H_21_N_5_Na_2_O_6_S_2_ |
| Source | Dystar | Sigma Aldrich |
| CASRN | 12221-69-1 | 3351-05-1 |
| Purity of dye (%) | 70–80 | Approximately 50 |
| Chemical structure | 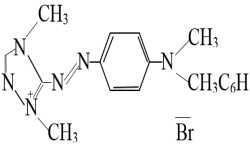 | 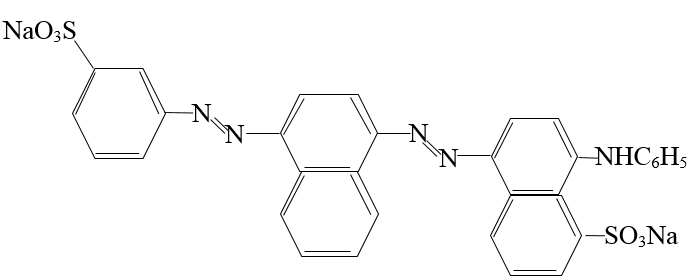 |

Note: CASRN, chemical abstracts survey registry number; BR, basic red; AB, acid blue.
