# Supplementary material for: Treatment of artificial wastewater containing two azo textile dyes by vertical-flow constructed wetlands
Source: Environ Sci Pollut Res Int. 2017 Dec 21;25(7):6870–89. doi: 10.1007/s11356-017-0992-0 (PMC5846842; doi:10.1007/s11356-017-0992-0)
Supplement: Supplementary file 3 — (DOCX 22 kb) [file 11356_2017_992_MOESM3_ESM.docx]

**Supplementary Material S3**

**Test of normality for plant and water quality variables**

The Shapiro-Wilk’s test ([Shapiro and Wilk, 1965](#_ENREF_61); [Razali and Wah, 2011](#_ENREF_54)) was used to investigate data normality. The corresponding results for the plant dimensions are shown in Table S3a. The lengths were approximately normally distributed for Wetlands 3, 4, 7, 8, 9, 10, 11, 12, 13, 14, 15, 16, 17 and 18 while the opposite was the case for Wetlands 5 and 6. The diameters were roughly normally distributed for Wetlands 3, 4, 6, 8, 9, 10, 11, 13, 14, 16, 17 and 18 while the reverse was noted for Wetlands 7, 12 and 15.

Regarding water quality variables in case of AB113, as shown in Table S3b, Wetlands 2, 3 and 9 were not normally distribution for all variables (dye concentration, pH, redox, EC, TSS, turbidity, DO, colour, COD, PO_4_-P, NH_3_-N and NO_3_-N). Wetland 13 had a normal distribution for redox, NH_4_-N and COD, while no normal distribution was noted for the dye concentration, pH, EC, TSS, turbidity, DO, PO_4_-P, NO_3_-N and colour. Wetland 17 was normally distribution for NO_3_-N, while the opposite was noted for dye concentration, pH, redox, EC, DO, TSS, turbidity, PO_4_-P, NH_4_-N, COD and colour.

In case of BR46, Wetland 1 was approximately normally distributed for COD, while the opposite was noted for dye concentration, pH, redox, EC, TSS, turbidity, DO, colour, NH_4_-H, NO_3_-N and PO_4_-P. Wetland 5 was linked to a normal distribution for pH and DO, while the opposite was obvious for dye concentration, redox, EC, TSS, turbidity, colour, NH_4_-N, NO_3_-N, PO_4_-P and COD. Wetland 7 was approximately normally distributed for DO, PO_4_-P and COD, while the reverse was the case for dye concentration, pH, redox, EC, TSS, turbidity, colour, NH_4_-N and NO_3_-N. Wetland 11 had a normal distribution for DO, while the opposite was recorded for dye concentration, pH, redox, EC, TSS, turbidity, colour, NH_4_-N, NO_3_-N, PO_4_-P and COD. Wetland 15 was normally distributed for PO_4_-P and COD, while the opposite was the case for dye concentration, pH, redox, TSS, turbidity, DO, colour, EC, NH_4_-N and NO_3_-N.

In case of the mixture between both dyes, Wetlands 4 and 12 were not normally distributed for all variables (dye concentration, pH, redox, DO, TSS, turbidity, EC, colour, NH_4_-N, NO_3_-N, PO_4_-P and COD). Wetlands 6 was normally distributed for PO_4_-P, while the opposite was found for the dye concentration, pH, redox, TSS, turbidity, EC, colour, DO, NH_4_-N, NO_3_-N and COD. The distribution was normal for Wetland 8 regarding redox, DO and PO_4_-P, while the opposite was found for dye concentration, pH, EC, TSS, turbidity, colour, COD, NH_4_-N and NO_3_-N. Wetland 10 was approximately normally distributed for dye concentration, while the reverse was discovered for pH, redox, EC, TSS, turbidity, DO, colour, COD, NH_4_-N, NO_3_-N and PO_4_-P. Wetland 14 was approximately normally distributed for redox, while the opposite was the case for dye concentration, pH, EC, TSS, DO, turbidity, colour, COD, NH_4_-N, NO_3_-N and PO_4_-P. Wetland 16 was approximately normally distributed for COD, NH_4_-N, NO_3_-N and PO_4_-P, while the contrary was clear for dye concentration, pH, redox, TSS, EC, DO, turbidity and colour. Wetland 18 had a normal distribution for dye concentration, turbidity, DO, COD, PO_4_-P, NH_4_-N and NO_3_-N. In contrast, the opposite was found for pH, redox, EC, TSS and colour.

**Table S3a** Test of normality for dimensions of *Phragmites australis* (Cav.) Trin. ex Steud. (Common Reed) related to different wetlands

| Characteristic | | | | Test of normality (if *p*>0.05, data are normally distributed; if *p* < 0.05, data are not normally distributed) | |
| --- | --- | --- | --- | --- | --- |
| Dye | Wetland number(s) | Number of stems | Length (mm) | | Diameter (mm) |
| BR46 | 5 | 68 | 0.042 | | 0.015 |
|  | 7 | 20 | 0.513 | | 0.000 |
|  | 11 | 17 | 0.720 | | 0.062 |
|  | 15 | 14 | 0.131 | | 0.025 |
| AB113 | 3 | 40 | 0.060 | | 0.402 |
|  | 9 | 15 | 0.082 | | 0.547 |
|  | 13 | 20 | 0.113 | | 0.937 |
|  | 17 | 10 | 0.335 | | 0.824 |
| Mix-ture of both dyes | 4 | 48 | 0.287 | | 0.364 |
|  | 6 | 55 | 0.004 | | 0.545 |
|  | 8 | 7 | 0.729 | | 0.970 |
|  | 10 | 10 | 0.592 | | 0.822 |
|  | 12 | 10 | 0.655 | | 0.004 |
|  | 14 | 16 | 0.548 | | 0.972 |
|  | 16 | 7 | 0.685 | | 0.996 |
|  | 18 | 9 | 0.122 | | 0.790 |

Note: BR, basic red; AB, acid blue.

**Table S3b** Test of normality for effluent water quality characteristics regarding general physical and chemical variables related to different wetlands

| Dye | Wetland number(s) | No. of samples | Test of normality (if *p*>0.05, data are normally distributed; if *p* < 0.05, data are not normally distributed) | | | | | | |  |
| --- | --- | --- | --- | --- | --- | --- | --- | --- | --- | --- |
|  |  |  | Dye concentration (mg/l) | pH (–) | | | Redox potential (mV) | | Dissolved oxygen (mg/l) |  |
| BR46 | 1 | 82 | 0.003 | 0.000 | | | 0.038 | | 0.000 |  |
|  | 5 | 82 | 0.002 | 0.916 | | | 0.004 | | 0.050 |  |
|  | 7 | 82 | 0.000 | 0.000 | | | 0.033 | | 0.564 |  |
|  | 11 | 81 | 0.000 | 0.000 | | | 0.000 | | 0.445 |  |
|  | 15 | 41 | 0.010 | 0.000 | | | 0.000 | | 0.014 |  |
| AB113 | 2 | 82 | 0.000 | 0.000 | | | 0.000 | | 0.000 |  |
|  | 3 | 82 | 0.000 | 0.000 | | | 0.000 | | 0.000 |  |
|  | 9 | 82 | 0.000 | 0.000 | | | 0.000 | | 0.000 |  |
|  | 13 | 81 | 0.000 | 0.000 | | | 0.110 | | 0.000 |  |
|  | 17 | 41 | 0.001 | 0.000 | | | 0.002 | | 0.000 |  |
| Mix-ture of both dyes | 4 | 82 | 0.015 | 0.000 | | | 0.000 | | 0.000 |  |
|  | 6 | 82 | 0.021 | 0.000 | | | 0.000 | | 0.000 |  |
|  | 8 | 82 | 0.000 | 0.001 | | | 0.053 | | 0.585 |  |
|  | 10 | 82 | 0.059 | 0.000 | | | 0.000 | | 0.024 |  |
|  | 12 | 81 | 0.000 | 0.000 | | | 0.000 | | 0.000 |  |
|  | 14 | 81 | 0.000 | 0.000 | | | 0.194 | | 0.000 |  |
|  | 16 | 41 | 0.000 | 0.000 | | | 0.014 | | 0.000 |  |
|  | 18 | 41 | 0.051 | 0.021 | | | 0.033 | | 0.411 |  |
| Characteristic | | | Total suspended solids (mg/l) | | Turbidity (NTU) | Electric conductivity (µS/cm) | | Colour (Pt Co.) | | |
| BR46 | 1 | 82 | 0.006 | 0.043 | | | 0.000 | | 0.000 |  |
|  | 5 | 82 | 0.002 | 0.000 | | | 0.000 | | 0.000 |  |
|  | 7 | 82 | 0.000 | 0.000 | | | 0.000 | | 0.000 |  |
|  | 11 | 81 | 0.009 | 0.042 | | | 0.000 | | 0.001 |  |
|  | 15 | 41 | 0.000 | 0.002 | | | 0.000 | | 0.005 |  |
| AB113 | 2 | 82 | 0.001 | 0.000 | | | 0.000 | | 0.000 |  |
|  | 3 | 82 | 0.006 | 0.000 | | | 0.000 | | 0.014 |  |
|  | 9 | 82 | 0.000 | 0.002 | | | 0.000 | | 0.005 |  |
|  | 13 | 81 | 0.012 | 0.004 | | | 0.000 | | 0.000 |  |
|  | 17 | 41 | 0.004 | 0.003 | | | 0.000 | | 0.000 |  |

**Table S3b** (continued)

| Characteristic | | | Total suspended solids (mg/l) | | Turbidity (NTU) | Electric conductivity (µS/cm) | | Colour (Pt Co.) | | |
| --- | --- | --- | --- | --- | --- | --- | --- | --- | --- | --- |
| Mix-ture of both dyes | 4 | 82 | 0.000 | 0.000 | | | 0.000 | | 0.000 |  |
|  | 6 | 82 | 0.000 | 0.000 | | | 0.000 | | 0.000 |  |
|  | 8 | 82 | 0.000 | 0.000 | | | 0.000 | | 0.000 |  |
|  | 10 | 82 | 0.000 | 0.000 | | | 0.000 | | 0.000 |  |
|  | 12 | 81 | 0.000 | 0.000 | | | 0.000 | | 0.000 |  |
|  | 14 | 81 | 0.000 | 0.000 | | | 0.000 | | 0.021 |  |
|  | 16 | 41 | 0.000 | 0.000 | | | 0.002 | | 0.015 |  |
|  | 18 | 41 | 0.026 | 0.062 | | | 0.000 | | 0.000 |  |
| Characteristic | | | Chemical oxygen demand (mg/l) | Ortho-phosphate-phosphorus (mg/l) | | | Ammonia-nitrogen (mg/l) | | Nitrate-nitrogen (mg/l) |  |
| BR46 | 1 | 30 | 0.357 | 0.018 | | | 0.026 | | 0.006 |  |
|  | 5 | 30 | 0.000 | 0.015 | | | 0.000 | | 0.000 |  |
|  | 7 | 30 | 0.210 | 0.324 | | | 0.000 | | 0.000 |  |
|  | 11 | 30 | 0.001 | 0.000 | | | 0.038 | | 0.009 |  |
|  | 15 | 30 | 0.318 | 0.151 | | | 0.012 | | 0.004 |  |
| AB113 | 2 | 30 | 0.000 | 0.000 | | | 0.021 | | 0.000 |  |
|  | 3 | 30 | 0.017 | 0.009 | | | 0.002 | | 0.000 |  |
|  | 9 | 30 | 0.035 | 0.000 | | | 0.006 | | 0.000 |  |
|  | 13 | 30 | 0.112 | 0.003 | | | 0.073 | | 0.002 |  |
|  | 17 | 30 | 0.000 | 0.001 | | | 0.000 | | 0.587 |  |
| Mix-ture of both dyes | 4 | 30 | 0.000 | 0.000 | | | 0.037 | | 0.000 |  |
|  | 6 | 30 | 0.000 | 0.079 | | | 0.000 | | 0.000 |  |
|  | 8 | 30 | 0.000 | 0.055 | | | 0.000 | | 0.000 |  |
|  | 10 | 30 | 0.000 | 0.000 | | | 0.008 | | 0.000 |  |
|  | 12 | 30 | 0.000 | 0.000 | | | 0.010 | | 0.000 |  |
|  | 14 | 30 | 0.000 | 0.000 | | | 0.016 | | 0.000 |  |
|  | 16 | 30 | 0.175 | 0.612 | | | 0.075 | | 0.109 |  |
|  | 18 | 30 | 0.990 | 0.149 | | | 0.092 | | 0.006 |  |

Note: BR; basic red, AB; acid blue
